# Recognition of Underwater Materials of Bionic and Natural Fishes Based on Blue-Green Light Reflection

**DOI:** 10.3390/s22249600

**Published:** 2022-12-07

**Authors:** Heng Jiang, Cuicui Zhang, Renliang Huang, Wei Qi, Rongxin Su

**Affiliations:** 1 State Key Laboratory of Chemical Engineering, Tianjin Key Laboratory of Membrane Science and Desalination Technology, School of Chemical Engineering and Technology, Tianjin University, Tianjin 300072, China; 2 School of Marine Science and Technology, Tianjin University, Tianjin 300072, China

**Keywords:** surface materials, underwater observation, blue-green light reflection, bionic fish recognition, machine learning

## Abstract

Thanks to the advantages of low disturbance, good concealment and high mobility, bionic fishes have been developed by many countries as equipment for underwater observation and data collection. However, differentiating between true and bionic fishes has become a challenging task. Commonly used acoustic and optical technologies have difficulty in differentiating bionic fishes from real ones due to their high similarity in shape, size, and camouflage ability. To solve this problem, this paper proposes a novel idea for bionic fish recognition based on blue-green light reflection, which is a powerful observation technique for underwater object detection. Blue-green light has good penetration under water and thus can be used as a signal carrier to recognize bionic fishes of different surface materials. Three types of surface materials representing bionic fishes, namely titanium alloy, carbon fiber, and nylon, are investigated in this paper. We collected 1620 groups of blue-green light reflection data of these three kinds of materials and for two real fishes. Following this, three machine learning algorithms were utilized for recognition among them. The recognition accuracy can reach up to about 92.22%, which demonstrates the satisfactory performance of our method. To the best of our knowledge, this is the first work to investigate bionic fish recognition from the perspective of surface material difference using blue-green light reflection.

## 1. Introduction

Recently, bionic fishes have been developed by many institutions as equipment for underwater environmental monitoring and ocean observation [1,2,3] since they look like real fishes and thus are not easily detected. In 1994, David Barrett of MIT developed the first bionic robotic fish in the world, the “RoboTuna” [4]. Later, they proposed an updated version of “RoboPike“ [5] through improvement in its mobility and stability. The University of Essex developed a bionic robotic fish named “G9” [6], which has been applied to marine pollution monitoring. In 1999, Beijing University of Aeronautics and Astronautics developed the first self-swimming bionic robot eel in China. Then, they improved it to create “SPC-II” [7], which was used for underwater archeological explorations of the Zheng Chenggong ancient warship site. The increased development of underwater bionic fishes has promoted studies of underwater target detection, fishery resource exploration, and so on. However, the detection and recognition of underwater bionic fishes has also been a challenging task due to their similarities to real fishes.

Acoustic “images” generated by active or passive sonars and visual images obtained from optical imaging systems are two types of media for underwater object detection. The former can usually detect objects from a long distance by determining the orientation, direction of motion, and structural information of targets based on sound transmission [8,9,10,11]. However, acoustic imaging methods are often criticized since they are easily disturbed by the marine environment, large target noise, and a low degree of automation. To improve the quality of acoustic images, several methods have been proposed. Yin et al. proposed a single-carrier multiuser receiver in underwater acoustic communications with strong multiple access interference (MAI), combining passive time-reversal (PTR) and direct-adaptation-based turbo equalization [12]. Zhou and Yang proposed a denoising method to suppress the noise interference in underwater acoustic signals for recognition [13]. Dunlop et al. used a multi-beam echosounder on a remotely operated vehicle to gather in situ information of abyssal benthopelagic assemblages and discern their distribution, behavior, and habitat associations [14]. Chen et al. proposed a network model (Efficientnet-S) adjusted by compound scaling based on the baseline model, to study active sonar target recognition on small samples. According to the experiments based on anechoic pool echoes, the proposed model achieves a recognition accuracy of more than 90% [15]. Wei et al. reviewed the literature from the previous decade on the use of these two types of imaging sonar in fish species identification, abundance estimation, length measurement and behavior analysis, as well as sonar imagery processing concerning fish, and proposed three challenges, including (1) the recognition of small fish forming dense aggregations; (2) species identification, which limits their use in species-specific studies; and (3) time-consuming massive data processing [16]. These studies are beneficial to the application of sonar in underwater object recognition. However, the acoustic images, which lack color and texture information, are still difficult to utilize for the detection of small volume targets. On the other hand, visual optical images with color and texture information have been used for the detection of underwater objects. To detect objects from optical images, several works have been proposed based on machine learning [17,18,19]. Liu and Liang proposed a method using background light estimation and improved adaptive transmission fusion [20]. Li et al. developed an underwater image enhancement method based on an underwater scene prior motived convolutional neural network, called UWCNN, which can be used to extract objects from videos frame by frame [21]. Although optical images can supply more abundant information than acoustic images, they are still challenged by several issues. For example, due to the physical properties of seawater, the color of underwater optical images is mainly blue and green with low contrast. Most of the reported methods recognize different underwater targets by shape structure, and to the best of our knowledge, there is no special method for recognizing real fishes and bionic fishes from the perspective of surface material difference. We aim to explore the feasibility of recognizing different materials underwater to provide new methods for recognition.

Blue-green light, the transmission window of sea water, which was discovered by Duntley [22], has been acknowledged as an important optical signal transmission medium for underwater observation. It has many advantages over the traditional white-light-based optical medium. It has stronger penetration and weaker attenuation in sea water, which leads to better performance when recognizing surface materials of different objects. Blue-green light has been widely used in many fields, such as underwater communication [23,24], sensing [25] and so on. However, no existing works have investigated its performance in the recognition of underwater bionic fishes from the perspective of surface material difference. In this paper, we will utilize blue-green light to classify materials, providing a novel idea for the recognition of real fishes and bionic fishes.

In this paper, the reflection characteristics defined by the parameter of reflection coefficient $R_{C}$ using the blue-green light of band 470.32–570.72 nm is utilized to classify different materials (titanium alloy, carbon fiber and nylon) representing bionic fishes and body parts of the real fishes (the abdomen, side, and back parts of sea bass and larimichthys crocea). We collected a dataset of 1620 groups of reflection coefficient R_C under varied environmental conditions, with differences in light propagation distance and salinity. Following that, three commonly used machine learning algorithms, including logistic regression (LR), back propagation (BP) neural network and support vector machine (SVM) are utilized for classification. To the best of our knowledge, this is the first work to investigate bionic fish recognition from the perspective of surface material difference based on blue-green light reflection.

The remainder of this paper is organized as follows. Section 2 describes the development of the whole system, including hardware construction, data collection, and data preprocessing. An evaluation of our method based on the experimental results is provided in Section 3. Finally, we conclude this paper with a description of future work in Section 4.

## 2. Methodology of Bionic Fish Recognition using Blue-Green Light Reflection

**Figure 1:**
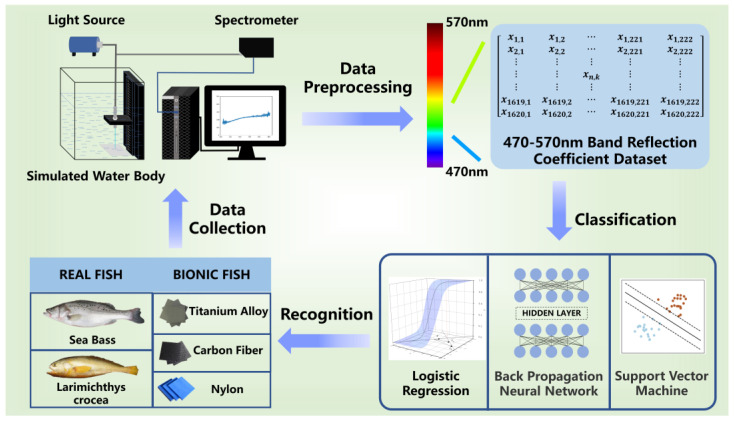
Schematic diagram of the whole process. The lower left shows the real fishes species and materials representing underwater bionic fishes we selected. On the upper left is the data acquisition device we built. The underwater Y-type optical fiber connects the spectrometer and the tungsten halogen lamp light source. The optical fiber head probing into the water is the transmitting end and receiving end of the light. The sliding platform fixing the optical fiber head is provided with a scale, which can read the light propagation distance. The data set we built is shown on the upper right, with a total of 1620 groups of data recorded to form the data set. The lower right are the machine learning methods we selected to verify the validity of the data set.

We conducted a feasibility study of the recognition of underwater materials of bionic and natural fishes by blue-green light in a small distance. Thus, we designed a recognition system using blue-green light reflection based on the theory that different material surfaces have different reflection characteristics. In this paper, we built optical hardware to collect reflectivity data of different materials and under different water environments to construct a dataset for recognition. Then, three traditional machine learning algorithms were utilized to verify the effectiveness of our method. The whole procedure is shown in Figure 1. Next, we will introduce each part of the system specifically.

### 2.1. System Development and Data Collection

**Figure 2:**
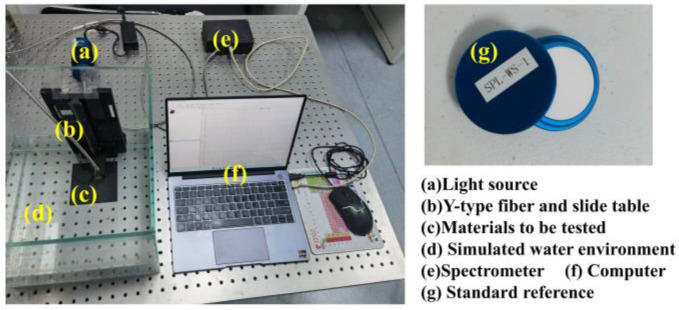
The hardware system developed for bionic fish recognition.

Optical data acquisition hardware was built to collect the light reflection data of different materials under different conditions in a 25 °C constant temperature chamber. The system is shown in Figure 2.

A tungsten-halogen light source (DH-2000-BAL, Ocean Optics, Inc., Dunedin, FL, USA) was used to provide stable light at a wavelength of 200–1000 nm. The light travels through the underwater Y-type optical fiber and is reflected by the surface of the material. The reflected light is transmitted to the spectrometer through the other side of the underwater Y-type optical fiber. The underwater Y-type optical fiber is inserted into the water. Spectrometer data are recorded using the “SpectraSuite” software. The reflection measurement mode of “SpectraSuite” software can take the reflection data of a standard reference object (SPL-WS-1, Hangzhou SPL Photonics Co., Ltd.) as the benchmark. The reflection coefficient of the material can be automatically generated according to this benchmark. We used a 30 cm long cube, glass water tank as a container.

If we directly use the reflected light intensity to distinguish different materials, the quality of collected data would be greatly affected by factors such as measuring distances and water environments. Therefore, we designed a method that can eliminate the influences of these factors. We first obtain the reflected light intensity of the standard reference object $I_{ref,\;\;std}$ and then the reflected light intensity $I_{ref}$ of the material to be measured under the same conditions. The reflection coefficient $R_{C}$ of the material can be obtained by dividing $I_{ref}$ by $I_{ref,std}$ as shown in Equation (3). Since the reflectivity of the standard reference object $R_{std}$ in Equation1 and the incident light intensity $I_{inc}$ remain stable, the reflection coefficient $R_{C}$ is proportional to the reflectivity R in Equation (2) of the material to be measured.

(1) $$R_{std}=\frac{I_{ref,std}}{I_{inc}}$$

(2) $$R=\frac{I_{ref}}{I_{inc}}$$

(3) $$R_{C}=\frac{I_{ref}}{I_{ref,std}}=\frac{R}{R_{std}}$$

Using this hardware system, we collected data of real and bionic fishes of different materials, under different light propagation distances and with different water conditions, respectively. Next, we will discuss about each kind of measuring situations in detail.

#### 2.1.1. Data Collection on Real and Bionic Fishes of Different Materials

Different body parts (including back, abdomen and side) of two real fishes (including sea bass and larimichthys crocea) were used in data collection. Additionally, three materials representing bionic fishes, including titanium alloy, carbon fiber, and nylon, were selected for data collection. Two real fishes and the three different materials representing bionic fishes are illustrated in Figure 1.

#### 2.1.2. Data Collection under Different Distances from the Light Source

**Figure 3:**
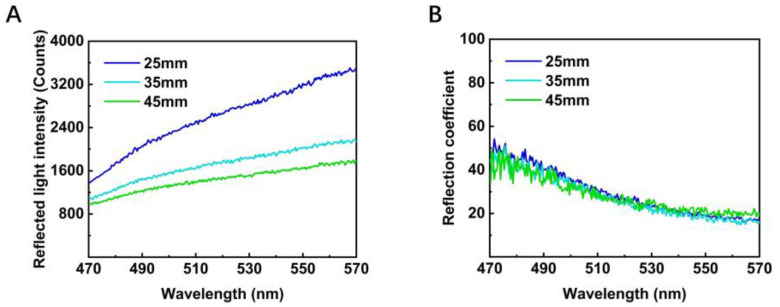
We chose nylon, one of the five materials, as a representative to show the difference in distance between the two data forms. (**A**) Light intensity $I_{ref}$ of nylon collected at different distances. (**B**) Reflection coefficient $R_{C}$ of nylon collected at different distances.

During data collection, we ensured that light was almost perpendicular to the surface of the target. The distance from the end of the optical fiber head to the surface of the material, i.e., the underwater propagation distance of the light, can be obtained using the scale on the slide. We performed experiments under the following three distances for each object to be measured: 25 mm, 35 mm and 45 mm, as shown in Figure 3.

#### 2.1.3. Data Collection under Different Physical Water Environments

**Figure 4:**
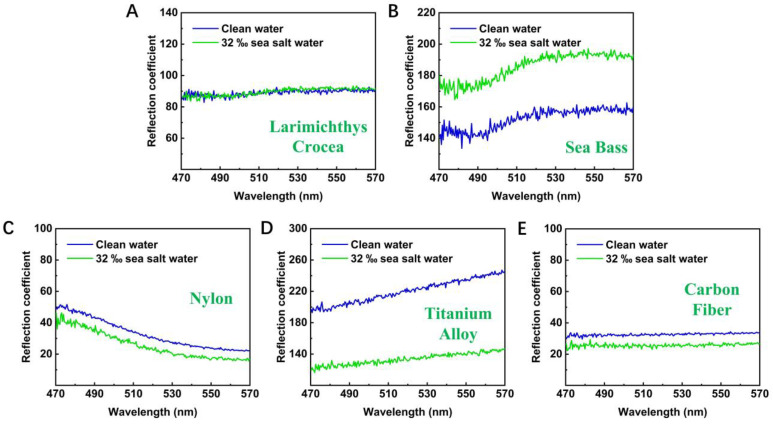
Average data ($R_{C}$) of various materials in clean water and water with salinity of 32‰, the type of material is marked in the subgraph. (**A**) Larimichthys crocea. (**B**) Sea bass. (**C**) Nylon. (**D**) Titanium alloy. (**E**) Carbon fiber.

We performed experiments under the following two water environments: clean water and 32‰ salinity simulated seawater. We averaged all the data of various materials under these two water environments and found that the shape of $R_{C}$ remains stable, but there was deviation along the longitudinal axis, as shown in Figure 4. This is because in order to ensure the recognition ability, we randomly selected 10 feature points on the surface of each material under each condition (repeating three times for each feature point) to collect data under each condition. Although the relative reflected light intensity of the same material is of great similarity, the difference still exists.

### 2.2. Bionic and Real Fish Recognition Based on Machine Learning

After data collection, all our data were intercepted in the range of 470.32–570.72 nm (blue-green band); then, we constructed a dataset comprised of 1620 groups of data with labels for classification, as shown in Table 1. We utilized machine learning algorithms to recognize different bionic and real fishes. Recently, machine learning has been greatly developed and received extensive attention thanks to the high development of computer hardware and the rapid improvement of the human ability to collect, store, transmit and process large data. Machine learning is advantageous in finding the inherent law of data to solve recognition and classification problems. In this paper, we utilize three commonly used machine learning algorithms, namely support vector machine (SVM), back propagation (BP) neural network and logistic regression (LR) to verify the effectiveness of our system and the developed dataset.

Support vector machine transforms the linearly inseparable data in the low dimension space to the high dimensional spaces [26,27,28]. It finds the hyperplane in the higher dimension to divide the data and utilizes the samples in the training set to locate the boundary. Several kernel functions are integrated in SVM, which make SVM more efficient and effective.

Linear regression combines various attributes of the data linearly to obtain the prediction function, then uses a monotone differentiable function to connect the true label of the classification task with the predicted value of the linear regression model, instead of just predicting categories. LR can achieve approximate probability prediction, which is useful for many tasks that need to use probability to assist in decision-making [29,30,31].

A back propagation neural network is a multilayer feedforward network trained according to error back propagation. When calculating the loss function, it is carried forward from input to output, while adjusting the weight and threshold backward. The BP neural network includes an input layer, hidden layer and output layer, the structure of which is similar to the neural structure of the human brain [32,33,34].

## 3. Experimental Results and Discussion

### 3.1. Experimental Setting

The experiments are performed using an AMD Ryzen 7 4800H (Advanced Micro Devices, Santa Clara, CA, USA) with Radeon Graphics and a memory of 16 GB. All the data were in txt form with 222 rows and 2 columns. We randomly selected 4/5 of samples (1296 groups) as training data and the remaining 1/5 data as test data. For verification, we used 5-fold cross validation.

The SVM algorithm has a hyperparameter C, which determines the regularization strength. A smaller C value represents a stronger regularization ability. In this work, we tried many values to determine the best hyperparameter C and found that the highest recognition accuracy can be achieved at a C of 5580. The kernel function we used was the radial basis function.

**Figure 5:**
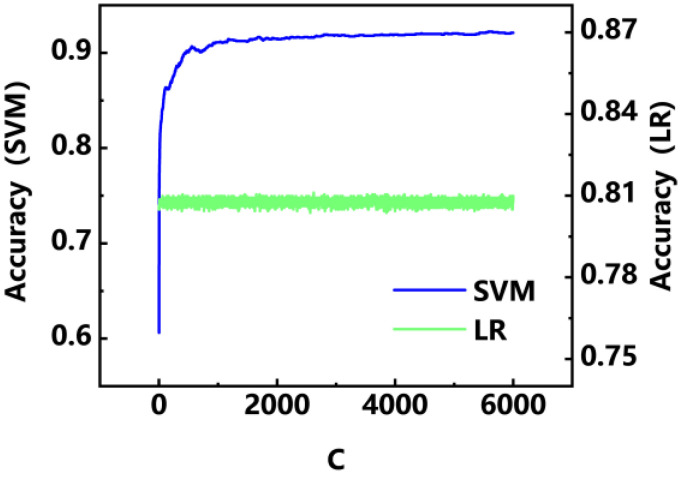
The hyperparameter C value and accuracy curve of SVM and LR, respectively.

**Figure 6:**
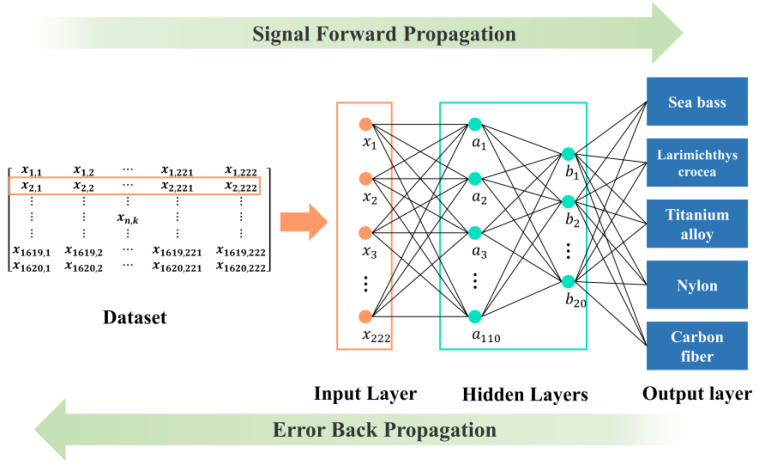
The BP neural network we constructed in this paper. The input data contains 222 data points. There are 110 neurons in the first hidden layer and 20 neurons in the second hidden layer. The final output is mapped to five categories.

Compared to SVM, the performance of LR is not as sensitive to the hyperparameter C, as shown in Figure 5. For LR, the solver was set as “lbfgs” and the regularization term was L2. The structure of the BP neural network we constructed in this paper is shown in Figure 6. The input data contain 222 data points. It normalizes the input data from [0, 500] to [0, 1]. The neural network adopts the stochastic gradient descent method. There are 110 neurons in the first hidden layer and 20 neurons in the second hidden layer. We set the learning rate η as 0.001. The final output is mapped to five categories.

### 3.2. Results and Analysis

The experimental results are shown in Table 2. The fit time denotes the time cost for one round of training in 5-fold cross validation. The recognition accuracy is defined by the ratio of the number of samples that the model correctly identifies in the test set to the total number of samples. We can see that SVM performs better than the other two algorithms in terms of both training time and accuracy. Its recognition accuracy can reach up to 92.22%, which demonstrates the effectiveness of our whole system. Its computational complexity is also very low since SVM requires less time for parameter training to learn the internal law of the data and easily finds the super plane to classify the data. In contrast, a BP network needs to train many parameters, and LR is more suitable for binary classification than multi-class classification.

**Figure 7:**
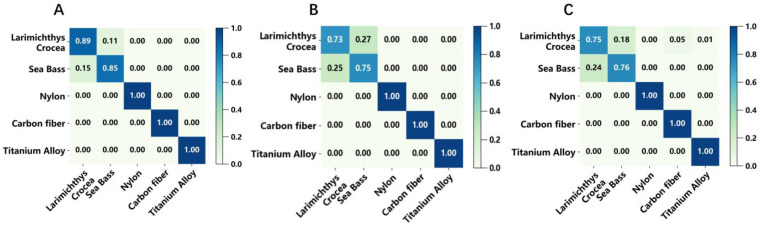
Confusion matrix of various methods. (**A**) SVM; (**B**) LR; (**C**) BP. The longitudinal axis is the real value and the transverse axis is the predicted value.

We drew the confusion matrices based on a certain recognition result (not 5-fold cross validation) to demonstrate the effectiveness of our method more comprehensively; the confusion matrix is normalized over the true conditions. As shown in Figure 7, the longitudinal axis represents the true value and the transverse axis is the predicted value. If the data of two real fishes are excluded, we can see that the recognition accuracy could reach 100% for the materials representing bionic fishes (titanium alloy, carbon fiber, nylon) in the SVM and LR, which validates our approach and the dataset well. Some other evaluation indicators including the precision, recall and F1-score are listed in Table 3. Precision is the proportion of correctly predicted instances among all instances predicted to be positive, recall is the proportion predicted to be correct among all positive categories, and the F1-score is twice the product of recall and precision divided by their sum. SVM still performs best under the evaluation of more indicators. We also tried to use the convolutional neural network method, as shown in Figure S1. In order to adapt the convolutional neural network method, we changed the data form to images. However, the accuracy of the convolutional neural network on the test set is 85.86%. Figure S2 shows the confusion matrix of results. When a large amount of training time and computing resources were spent, compared with traditional methods, this method did not show advantages.

## 4. Conclusions

In this work, we proposed a novel blue-green light reflection method to recognize different materials underwater, providing a new idea for underwater bionic fish recognition. Taking the reflected light intensity of the standard reference object as the benchmark, the reflection coefficients of different materials are proportional to their own reflectivity. The processed data are distributed between 470.32 nm and 570.72 nm and are composed of 222 data points. By taking advantage of the reflected light intensity of the standard object, the reflection coefficient data are less affected by distance and water quality. We collected a total of 1620 groups of data in five categories (including different parts of two real fishes and three kinds of bionic fish materials) under different environmental conditions, such as distances and salinities. Following this, the effectiveness of our method was verified with the following three machine learning algorithms: support vector machine, logistic regression and back propagation neural network. Each algorithm had a high accuracy, and the highest accuracy was obtained by the support vector machine, of 92.22%.

Matteoli et al. proposed a subspace-based approach to investigate underwater material discriminability. Synthetic test data were generated using a simulator: eight different objects (e.g., fiberglass, tin, neoprene, aluminum) were simulated as being submerged 10 m deep within the water column, while four out of eight objects were correctly recognized with an accuracy of more than 75% [35]. Liu et al. designed a method of matching the same underwater object in acoustic and optical images. The distance between the camera or sonar and the target object (e.g., shellfish, sea urchins, stone) was 60 cm, and the recognition accuracy was about 90% [36]. It can be seen that the shape of the targets recognized using the method with high recognition accuracy is quite different; the method with a long target recognition distance has low recognition accuracy.

The recognition method based on the differences of underwater bionic fish surface materials in the blue-green band has great application prospects. When applied to underwater robots, this method can effectively identify different underwater targets with high similarity, which is of great significance for underwater target detection, fishery resource exploration, and other research fields. In the future, we would like to collect more data of real fishes and bionic fish materials and improve hardware systems by using lasers for better classification. We also plan to use more sophisticated deep learning methods to improve the accuracy of recognition.

## Figures and Tables

**Table 1:** Dataset composition (number of various samples under different conditions).

	Sea Bass	Larmichthys Crocea	Titanium Alloy	Carbon Fiber	Nylon
25 mm distance in clean water	90	90	30	30	30
35 mm distance in clean water	90	90	30	30	30
45 mm distance in clean water	90	90	30	30	30
Total in clean water	270	270	90	90	90
Total in two water environments	540	540	180	180	180

**Table 2:** Performance of each model in 5-fold cross validation.

Model	Total	Fit Time(s)	Test Accuracy (%)
SVM	324	0.09	92.22
LR	324	1.39	81.11
BP	324	37.07	84.62

**Table 3:** Performance of each model in a certain recognition result.

	SVM			LR			MLP		
	Precision	Recall	F1-Score	Precision	Recall	F1-Score	Precision	Recall	F1-Score
Larmichthys Crocea	0.878	0.886	0.882	0.792	0.737	0.764	0.842	0.746	0.791
Sea bass	0.863	0.854	0.858	0.716	0.771	0.742	0.792	0.833	0.812
Nylon	1	1	1	1	1	1	1	1	1
Carbon Fiber	1	1	1	1	1	1	0.774	0.75	0.762
Titanium Alloy	1	1	1	1	1	1	0.975	1	0.987

## Data Availability

Not applicable.

## Supplementary Materials

The following supporting information can be downloaded at: https://www.mdpi.com/article/10.3390/s22249600/s1, Figure S1: The convolution neural network structure we designed; Figure S2: Confusion matrix of convolutional neural network on test set.
