# Supplementary material for: Recognition of Underwater Materials of Bionic and Natural Fishes Based on Blue-Green Light Reflection
Source: Sensors (Basel). 2022 Dec 7;22(24):9600. doi: 10.3390/s22249600 (PMC9781537; doi:10.3390/s22249600)
Supplement: Supplementary file 1 [file sensors-22-09600-s001.zip › sensors-2018642-supplementary.pdf]

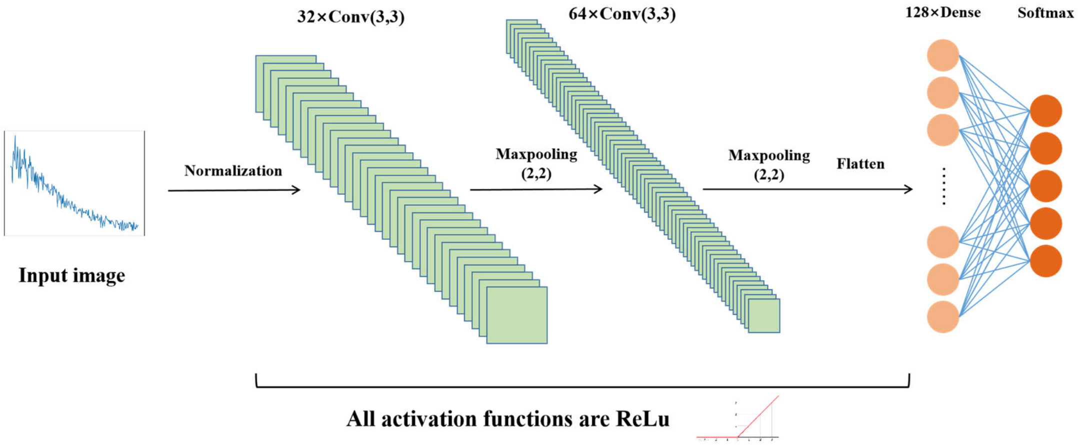

Figure S1. The convolution neural network structure we designed. The first layer is used for data normalization, the second layer is a convolution layer including 32 convolution cores, the third layer is a pooling layer, the fourth layer is a convolution layer including 64 convolution cores, the fifth layer is a pooling layer, the sixth layer is a flattening layer, the seventh layer is a full connection layer including 128 nodes, and the eighth layer is an output layer.

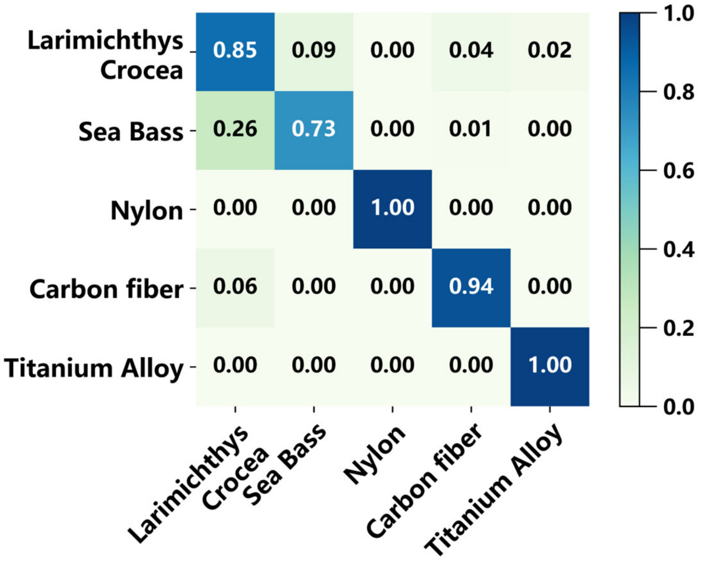

Figure S2. Confusion matrix of convolutional neural network on test set (normalized over the true conditions).
